# Proteomics and Phospho-Proteomics Profiling of the Co-Formulation of Type I and II Interferons, HeberFERON, in the Glioblastoma-Derived Cell Line U-87 MG

**DOI:** 10.3390/cells11244068

**Published:** 2022-12-15

**Authors:** Dania Vázquez-Blomquist, Anette Hardy-Sosa, Saiyet C. Baez, Vladimir Besada, Sucel Palomares, Osmany Guirola, Yassel Ramos, Jacek R. Wiśniewski, Luis Javier González, Iraldo Bello-Rivero

**Affiliations:** 1 Pharmacogenomic Group, Department of System Biology, Biomedical Research Division, Center for Genetic Engineering and Biotechnology, Havana 10600, Cuba; 2 KU Leuven, 3000 Leuven, Belgium; 3 Département de Neurosciences, Université de Montréal, Montréal, QC H2L0A9, Canada; 4 Proteomics Group, Department of System Biology, Biomedical Research Division, Center for Genetic Engineering and Biotechnology, Havana 10600, Cuba; 5 Biochemical Proteomics Group, Department of Proteomics and Signal Transduction, Max-Planck Institute of Biochemistry, 82152 Munich, Germany; 6 Clinical Assays Direction, Center for Genetic Engineering and Biotechnology, Havana 10600, Cuba

**Keywords:** HeberFERON, interferon, glioblastoma, U-87 MG, protein phosphorylation, phospho-proteomic

## Abstract

HeberFERON, a co-formulation of Interferon (IFN)-α2b and IFN-γ, has effects on skin cancer and other solid tumors. It has antiproliferative effects over glioblastoma multiform (GBM) clones and cultured cell lines, including U-87 MG. Here, we report the first label-free quantitative proteomic and phospho-proteomic analyses to evaluate changes induced by HeberFERON after 72 h incubation of U-87 MG that can explain the effect on cellular proliferation. LC-MS/MS, functional enrichment and networking analysis were performed. We identified 7627 proteins; 122 and 211 were down- and up-regulated by HeberFERON (fold change > 2; *p* < 0.05), respectively. We identified 23,549 peptides (5692 proteins) and 8900 phospho-peptides; 523 of these phospho-peptides (359 proteins) were differentially modified. Proteomic enrichment showed IFN signaling and its control, direct and indirect antiviral mechanisms were the main modulated processes. Phospho-proteome enrichment displayed the cell cycle as one of the most commonly targeted events together with cytoskeleton organization; translation/RNA splicing, autophagy and DNA repair, as represented biological processes. There is a high interconnection of phosphoproteins in a molecular network; mTOR occupies a centric hub with interactions with translation machinery, cytoskeleton and autophagy components. Novel phosphosites and others with unknown biological functionality in key players in the aforementioned processes were regulated by HeberFERON and involved CDK and ERK kinases. These findings open new experimental hypotheses regarding HeberFERON action. The results obtained contribute to a better understanding of HeberFERON effector mechanisms in the context of GBM treatment.

## 1. Introduction

Interferons (IFNs) are cytokines with pleiotropic actions including antiviral and growth-inhibitory effects. These cytokines are the first line of defense against viral infections and have important roles in immunosurveillance for malignant cells [1]. Signaling crosstalk between IFN-α/β and -γ to induce stronger responses has been described [2]. IFNs type I and II transduce the signals after receptor binding through multiple cascades which include: JAK/STAT, phosphoinositide 3-kinase (PI3K)/mTOR and MAPKs [1,3]. Much activation/deactivation of these cascades occurs through phosphorylation/dephosphorylation reactions. The last two cascades normally activate survival signals, but in IFN response they function in such a way that the outcome is anti-proliferation, apoptosis, cell cycle arrest, or other processes leading to the decrease of cell growth and, eventually, their death [4]. Cells integrate information from multiple signaling pathways for a final proliferation outcome, so deciphering molecular mechanism is truly challenging.

The co-formulation of IFN-α2b and IFN-γ in synergic proportions, known as HeberFERON, has been approved for the treatment of skin baso-cellular carcinoma but has also been used off-label for other types of cancers, including glioblastoma [5]. On the other side, due to its strong antiviral effect, HeberFERON has been more recently assayed in COVID-19 patients [6] and was included in the Cuban medical protocol for COVID-19 treatment [7]. 

GBM is the most lethal tumor of the brain with an average of 14 months of overall survival (OR) [8]. Different approaches have been assayed for GBM treatment with modest results in terms of OR, even though improvement in the quality of life of patients has been reported [9]. The use of HeberFERON in high-grade gliomas (one anaplastic astrocytoma and nine GBM) in a compassionate study prolonged the survival of patients to a mean of 34 ± 14 months since diagnosis. The quality of life for all patients improved and the Karnofsky Performance Scale (KPS) score and muscular power was improved in 50% of patients [10]. We have demonstrated an antiproliferative response of a panel of 34 glioblastoma-derived clones and several in vitro cell lines to HeberFERON [11]. Glioblastoma-derived cell line U-87 MG treated with HeberFERON has been chosen as a model.

Cell signaling is the biochemical process by which cells respond to perturbations in their environment, extracellular stimuli, or intracellular cues where the engagement of IFNs to their receptors would be one of those perturbations. Treatment of U-87 MG with HeberFERON causes an antiproliferative effect with a decrease in cell numbers in the treated culture [12]. Changes in overall quantities of transcripts after 72 h of treatment have already been studied using a microarray chip showing that the cell cycle is one of the most affected processes by the co-formulation [12]. Protein quantities and protein modification changes are also important ways to understand the biological effect of HeberFERON. 

Cell signaling occurs through protein post-translational modifications, including phosphorylation [13]. Protein phosphorylation at serine, threonine and in <1% tyrosine is one the most common post-translational modifications. Protein phosphorylation controls protein activity, localization and protein complex formation [13,14]. In humans, 518 protein kinases control phosphorylation. Dysregulation of kinase signaling pathways is commonly associated with various cancers [15]. The understanding of the molecular mechanisms HeberFERON uses to achieve the anti-proliferation state on this glioblastoma-derived cell at different levels of regulation could have an impact on the control of this type of tumor. 

Here we report the proteome and phospho-proteome changes of the U-87 MG cell line after the treatment with HeberFERON for 72 h. 

## 2. Materials and Methods

### 2.1. Preparation of Samples for Proteomic Experiment

U-87 MG cell line (ECACC 89081402) was grown in EMEM with 1.5 g/L of NaHCO_3_, 2 mM of glutamine (Sigma, St. Louis, MO, USA), 50 µg/ mL of gentamicin sulfate (Sigma, St. Louis, MO, USA) and 10% of fetal bovine serum (Capricorn Scientific GmbH, Ebsdorfergrund, Germany). Ten million cells per condition were treated with HeberFERON (IFNα2b + IFNγ) at the concentration equivalent of IC50 (4000 IU/mL) [12], for 72 h. Three independent replicated cultures were treated or not (control) in the experiment. After washing the pellet with PBS, cells were resuspended in 6 M guanidine hydrochloride, 100 mM buffer Tris-HCl and 10 mM DTT in presence of proteases and phosphatase inhibitors. Cells were vortexed for 10 s and kept shaking for 15 min, incubated for 2 h at 37 °C and later made up to 25 mM iodoacetamide, kept dark for 20 min at 25 °C and centrifuged for 3 h at 60,000× *g*. Proteins were processed by MED-FASP [16] with consecutive digestion using Lys-C and trypsin as described previously [17]. A small fraction of peptides from each digestion was desalted separately and later analyzed via LC-MS/MS for non-phospho-peptides identification and further proteome differential expression analysis. Phospho-peptides were enriched from each Lys-C and Trypsin-derived digestion by using TiO_2_ beads with a 4:1 ratio (mg beads:mg peptides) as described previously [18].

### 2.2. LC-MS/MS and Identification of Peptides and Phospho-Peptides

Chromatographic runs for phospho-peptides and non-phospho-peptides were in a Reprosil-Pur 1.9 µm C18 (Dr. Maisch, Ammerbuch, Germany) home-made column (75 μm ID, 20 cm length), 120 min gradient, starting at 5% B (0.5% acetic acid in 80% acetonitrile), up to 30% B in 95 min, then increasing to 60% B in 5 min and up to 95% in 5 min more. NanoLC was coupled to a Q-exactive HF mass spectrometer; the mass range 300–1650 *m*/*z* was scanned using data-dependent acquisition. Each mass spectrum obtained at 60,000 resolution (20 ms injection time) was followed by 15 MS/MS spectra (28 ms injection time) at 15,000 resolution.

A label-free approach for the identification of peptides and proteins was usesd. This was based on the match-between-runs procedure by using Maxquant (v1.6.2.10) and partial oxidation (M), deamidation (NQ), N-terminal acetylation (proteins) and phosphorylation (STY) were considered as variable modifications and propionamide cysteine as fixed modification. Database searching in UniprotKB (2018) was performed with target-decoy. Alignment of chromatographic runs was allowed with default parameters (20 min time window and matching of 0.7 min between runs). Proteins and phospho-peptides were only considered when detected in at least two replicates in any of the groups with a false discovery rate (FDR) of 0.01. Statistical analysis for the relative quantification of phospho-peptides (class I sites with the probability of modification higher than 0.75) changing more than two-fold (*p* < 0.05) was performed by Perseus (v1.6.2.2) after filtering for two valid values in at least one group [19]. Student’s *t*-test was used for selection of proteins and phospho-peptides differentially expressed for *p* < 0.05 with FDR correction. Peptides with phosphorylation levels changing in the same direction as at the protein level were discarded.

### 2.3. Bioinformatic Enrichment Analysis

Enrichr [20] and Toppfun [21] were used for protein enrichment analysis with a *p*-value cutoff of 0.05 and Homo sapiens as a background. Additionally, DAVID bioinformatic resource was also used to compare enrichment using backgrounds Homo sapiens or the identified proteins in this experiment (accessed 2 December 2022) [22]. In DAVID, a modified Fisher’s exact Test (EASE score) is adopted to measure the gene enrichment in annotation terms using the default thresholds. KEA2 [23], KSEA [24] and iPTMNet [25] were used for Kinase-Substrate Enrichment Analysis. KEA2 web tool (https://www.maayanlab.net/KEA2/, accessed on 3 October 2022) computes a Fisher Exact Test to distinguish significantly enriched kinases (*p*-values lower than 0.05), through statistical analysis. KEA is focused on substrate overrepresentation rather than kinase scoring. KSEA app (https://casecpb.shinyapps.io/ksea/, accessed on 3 October 2022) is based on a z-score transformation assuming that the resulting scores were normally distributed. The score of a kinase is based exclusively on the collective phosphorylation status of its substrates and a negative score has substrates that are generally dephosphorylated with the test group. *p*-value is automatically calculated by the program by assessing the one-tailed probability of having a more extreme score than that measured, followed by a Benjamini-Hochberg FDR correction for multiple hypothesis testing. KSEA app is based in K–S database that included PhosphoSitePlus (PSP) information and relationships predicted by NetworKIN. iPTMnet based on a set of curated databases such as PSP (http://www.phosphosite.org, accessed on 3 October 2022), and PhosphoEML (http://phospho.elm.eu.org, accessed on 3 October 2022), annotates experimentally observed post-translational modifications. Sequence logos for those enriched motifs from phosphorylation sites with a *p* < 0.05 between control and experimental conditions were generated using WebLogo 3.7.4 (http://weblogo.threeplusone.com/, accessed on 3 October 2022). Phosphosite Motif analysis was also carried out using the Phosphosite algorithm [26]. Most of the resources, except DAVID, were accessed 3 October 2022. PSP (http://www.phosphosite.org, accessed on 3 October 2022) was used for additional information on phosphosites. 

### 2.4. PPI Analysis

All identified phosphoproteins were represented in a network context. STRING database (https://string-db.org/) (accessed on 24 March 2022) was used to identify functional and physical associations between proteins. Only curated knowledge and experimental evidence were used as the source of interaction data in such analysis and the confidence score was fixed at 0.4. The protein–protein interaction network was visualized using Cytoscape software (v.3.8.2); the MCL algorithm was used to identify clusters of tightly connected proteins within the network. Bisogenet (version 3.0.0) [27] was used to construct small networks with proteins participating in relevant biological processes.

## 3. Results

### 3.1. General Proteomic and Phosphoproteomic Results

**Figure 1:**
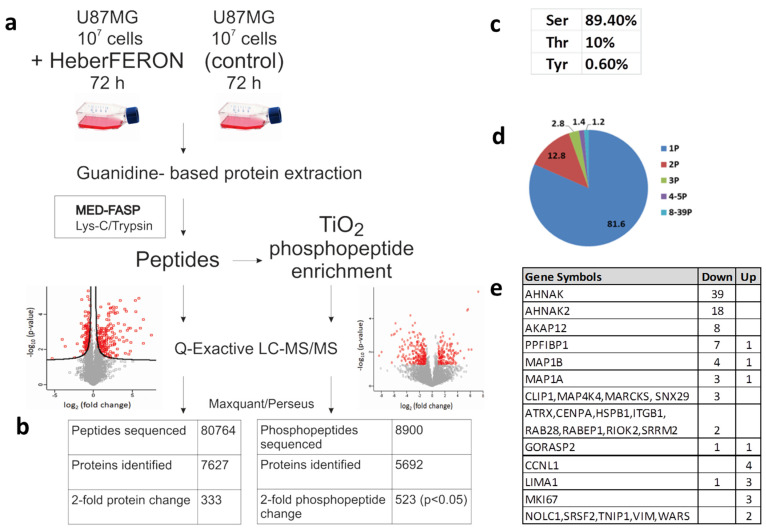
Experimental workflow and data processing for the identification of non-phospho-peptides and phospho-peptides regulated by HeberFERON. (**a**) Experimental workflow for sample processing, MS and data analysis (**b**) Comparison of identified non-phospho-peptides, phospho-peptides and proteins (**c**) Distribution of phosphorylated amino acids in % (**d**) Number of phosphorylation sites per protein represented in a pie diagram as % (**e**) Proteins with 2 or more different phosphosites changing phosphorylation. Up and Down represent the increment or decrement of the phosphorylation after HeberFERON treatment in the untreated control. Gene symbols are represented.

In this work, we studied protein and phosphorylation changes that could be involved in the antiproliferative effect of HeberFERON over the glioblastoma U-87 MG cell line. To survey proteome and phospho-proteome we treated U-87 MG with HeberFERON for 72 h and enriched phospho-peptides from proteomes that were digested following the MED-FASP processing (Figure 1a). After phospho-peptides enrichment with TiO_2_, we identified 23,549 peptides (derived from 5692 proteins), 38% (8900) of them were phospho-peptides; 6415 of these phospho-peptides were selected for statistical comparison among replicates. Considering a fold change higher than 2 for those Class I phospho-peptides (with a localization probability higher than 0.75), 523 phospho-peptides (*p* < 0.05) were identified, corresponding to 359 proteins (Table S1). A small fraction before phospho-peptide enrichment was separated for differential protein analysis. After LC-MS/MS 80764, peptides from around 7627 proteins were detected and around 6000 of them were selected for statistical processing. We found 333 proteins regulated by HeberFERON with *p* < 0.05 and a fold change higher than 2; 122 were down-regulated and 211 were up-regulated (Figure 1b, Table S2). 

The distribution of pS, pT and pY in phosphosites were 89.4, 10 and 0.6%, respectively (Figure 1c). Peptides were predominantly singly and doubly phosphorylated, but a few proteins showed multi-phosphorylation of up to 39 sites (Figure 1d,e). We identified 163 phosphosites increasing phosphorylation (from 141 proteins) and 345 phosphosites decreasing phosphorylation (from 225 proteins). 

### 3.2. Enrichment Analysis

**Figure 2:**
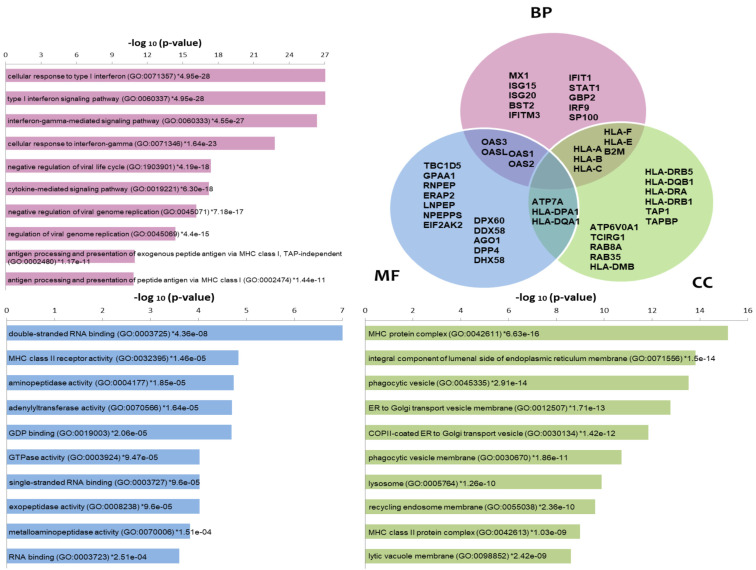
Enrichment analysis of proteomic data using the Enrichr tool. The most commonly enriched terms −log10 (*p*-values)) in the list of differentially expressed proteins are shown in the categories Biological Processes (BP), Molecular functions (MF) and Cellular components (CC) as bars. A Venn diagram is also used to indicate some of the proteins included in the categories as well as those common among categories. Gene symbols are used in the Venn diagram.

Enrichr tool highlighted biological processes (BP) enriched in the list of differentially expressed proteins, such as IFNs signaling and cellular response to IFNs, negative regulation of viral life cycle or genome replication and antigen processing and presentation via MHC (Figure 2). These processes are represented by classical encoding Interferon stimulated genes (ISG) such as B2M, GBP2, IFIT1, IFITM3, ISG15, ISG20, IRF9, HLA-A, HLA-B, HLA-C, HLA-F, HLA-E, MX1, OAS1, OAS2, OAS3, OASL, SP100 and STAT1. Among the molecular functions (MF) enriched in that list, we found those related to the double-stranded & single-stranded RNA binding (ex. RIG1 coding genes (DDX58), PKR (EIF2AK2)) and GDP binding, MHC class II receptor & aminopeptidase/peptidase/GTPase activities. In Cellular components, MHC class I and II complexes, protein complex components from the endoplasmic reticulum (ex.TAP1, TAPBP), Golgi, phagocytic vesicles, lysosome and recycling endosome (ex.RAB8A, RAB35) stand out as the enriched terms. Several proteins are shared among these categories, which was also represented through a Venn diagram (Figure 2). Pathways enrichment by Toppfun also pointed to Interferon signaling pathways as the most statistically represented (Table S3). Antigen processing and presentation, adaptive and innate immune systems and the antiviral mechanism of IFN-stimulated genes are well represented. Additional pathways comprised the signaling pathway from G-protein families (which includes kinases encoded by PRKCA and RPS6KA3); RAB GEFs exchange GTP for GDP on RABs membrane trafficking and Proteosome (more specifically Immunoproteasome components). We made an additional analysis using DAVID resource comparing Homo sapiens and experimentally identified proteins as backgrounds for enrichment analysis, which corroborated the same processes and pathways as those most enriched in the differentially expressed protein list. This exploration also showed new, interesting terms such as autophagy and cytoskeleton-dependent intracellular transport as biological processes and cytoskeletal adaptor activity and kinase among the molecular functions. As part of the list of kinases we found EIF2AK2/PKR, PRKCA, RPS6KA3, PRKAR2B, PRKAR2A, PRKD2 and NEK9 (Table S4). Network analysis of the proteome data showed highly interconnected biomarkers from STAT1/STAT2 to antiviral (Figure S1a) and MHC processing/presentation and immunoproteasome (Figure S1b) components. 

**Figure 3:**
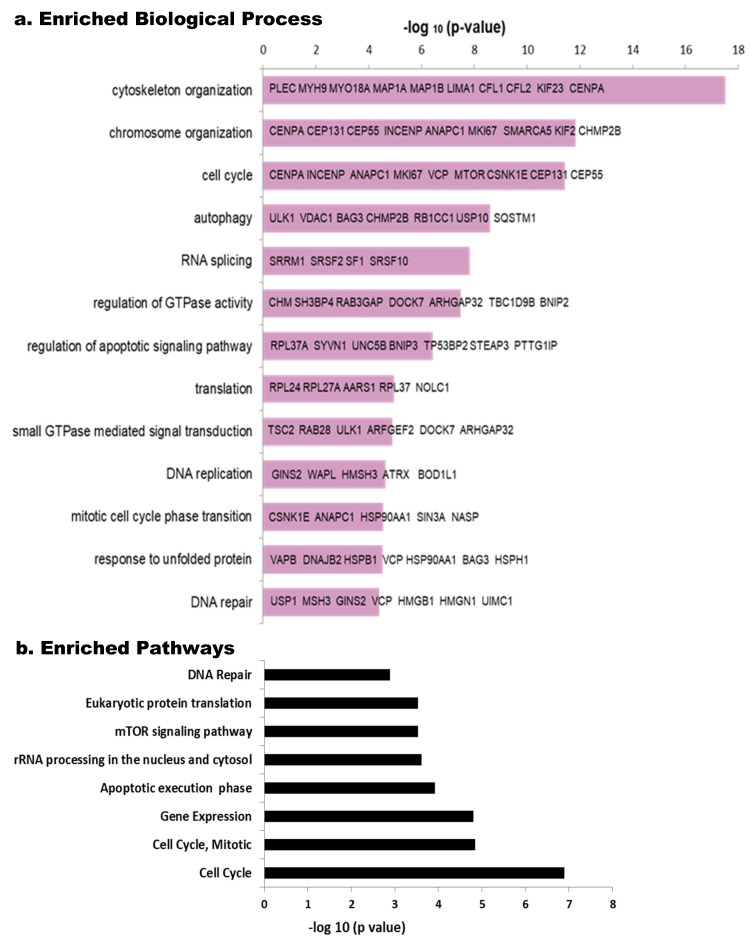
Enriched Biological processes and Pathways in phospho-proteomic data using the Toppfun tool. The reciprocal of Log (p) is tabulated for each Biological Process (**a**) and Pathway (**b**). Some of the principal components of the Biological Processes are shown in bars, as gene symbols.

Phosphoproteins enrichment analysis using the Toppfun tool detected relevant biological processes (Figure 3a). Among the most commonly represented, we found cytoskeleton organization, followed by chromosome organization and cell cycle, while others such as autophagy, RNA splicing, regulation of GTPase activity and regulation of apoptotic signaling pathway, translation, DNA replication, DNA repair, small GTPase mediated signal transduction and response to unfolded protein are shown with differential enrichment according to their −log (*p*-value) (Figure 3a). Looking at the pathways using the same tool, Cell Cycle (more specifically Mitotic) is among the most relevant paths, as well as, those related to Gene Expression, rRNA processing, apoptosis and DNA repair (Figure 3b). mTOR signaling pathway seems to be regulated by HeberFERON changing phosphorylation levels of different phosphoproteins from this pathway. DAVID analysis also pointed to these processes and pathways as the most enriched (Table S5). 

### 3.3. Analysis of Significant Kinases through Regulated Phosphosites

**Figure 4:**
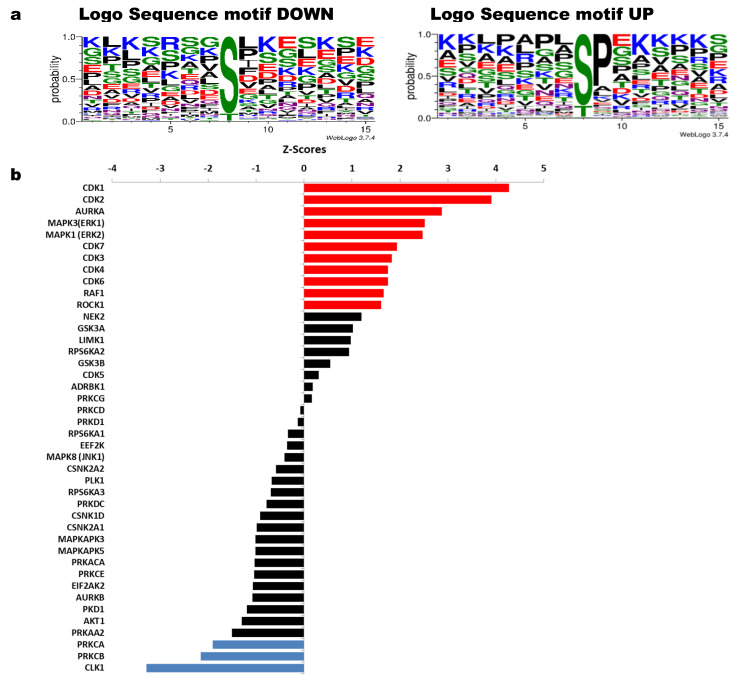
Sequence motifs logo and kinases analysis from phosphosites with increased or decreased phosphorylation after U-87 MG treatment with HeberFERON. (**a**) Logo Sequence motifs found for phosphosites that increased (UP) and decreased (DOWN) phosphorylation using WebLogo 3.7.4 (**b**) Analysis of kinases using the KSEA tool taking into account phosphosites which are statistically regulated; red (for sites with increased phosphorylation) and blue (for sites with decreased phosphorylation) for kinases with Z scores with statistically significance *p* < 0.05; in black, other kinases with *p* > 0.05 were also tabulated according to their Z score. Gene symbols are displayed.

Figure 4 shows sequence motifs with a LogoSequence graph for phosphosites that increased (UP) or decreased (DOWN) phosphorylation with HeberFERON (Figure 4a). Analysis of these motifs was carried out using the Motif-All algorithm in PSP (Table S6). Motifs with the highest probability for phosphosites increasing phosphorylation look like X-S-P-X-K. This Proline-directed phosphosite is typical for CDK and ERK proteins. To a lesser extent, mTOR motif like X-T-P-X-X-S-K was also predicted. KSEA program (Figure 4b) similarly pointed to CDK1, CDK2, MAPK3/K1 (alias ERK1/2) and CDK3/4/6/7, as those kinases with the highest z-scores based on the collectively increased phosphorylation status of their phosphosites with a *p* < 0.05. This program showed other kinases such as AURKA, RAF1 and ROCK1. On the other hand, motifs with the highest probability for phosphosites decreasing phosphorylation are more variable but we observed a general motif such as RK-RK-K-S-FLV-KR/X, corresponding to protein kinases (PK) ABC family. KSEA showed kinases with the lowest z-Scores based on the decreased phosphorylation status of their phosphosites including PRKCA (PKA) and PRKCB (PKB). Additional kinases with z-scores from 0 to |1| could also be responsible for phosphosites modification. These are the cases for NEK2, RPS6KA2 and GSK3B regulating phosphosites with phosphorylation increases and AKT1, PKD1, AURKB, EIF2AK2, PRKCE, PRKDC, PLK1, EEF2K, RPS6KA1/A3 for phosphorylation decreases. 

Table 1 listed some of the most interesting phosphosites on substrates regulated by enriched kinases from KSEA analysis. Several phosphosites were obtained after the analysis with KEA2 and/or iPTMNet programs. Following kinase predictions, most of the phosphosites changing phosphorylation levels were CDK1/CDK2/PRKCA/PRKCB substrates and some of them were also regulated by ERK1 & ERK2, AURKA, or CDK3/4/6/7. We found regulated phosphosites in proteins with important roles in cell cycle and proliferation (i.e., RB1_S807, MKI67_S584, INCENP_S421, CENPA_S19, MTOR_S1261), cytoskeleton organization (i.e., MAP1B_T1788, MARCKS_S27, MARCKS_S29), translation (EIF5B_S222, RPL12_S38), DNA repair and autophagy (i.e., BRCA1_S114, RAD9A_S328, RFC1_T506, SQSTM1_S272, MTOR_S1261) and in multifunctional proteins like NPM1/B23, HSPB1/HSP27, AKAP12 and AHNAK. Substrates of RAF1 & ROCK1 (VIM_S420 and CFL1_S3) and GSK3B (several sites on MAP1B, CLIP1/2) were regulated. PLK1, AURKB, AKT1, EIF2AK2 (PKR), RPS6KA3, PKD1 and other PKC kinases are associated with the decreased phosphorylation in phosphosites on HSPB1/HSP27, mTOR and NPM1/B23. 

There are numerous regulated phosphosites that have not been described or that have been described with no associated kinases for phosphorylation. Table 2 shows some of these sites obtained after iPTMNet analysis, divided by Biological Pathways. Several of these phosphosites are on proteins participating in Cytoskeleton organization and Cell Cycle (i.e., MAP1A and MAP1B, MYH9, MYO18A and 9B, LIMA1, MARCKS, KIF23 and PCM1), Autophagy (i.e., RAB7A and RB1CC1), Translation (i.e., EIF4G1 & G3), RNA Splicing and on multifunctional proteins such as AHNAK, AHNAK2 and AKAP12, which show several phosphosites decreasing phosphorylation. We found a decreased phosphorylation in the mTOR signaling member, GSK3B_S25 and increased phosphorylation in the new phosphosite IFNAR1_T328.

### 3.4. Network Analysis

**Figure 5:**
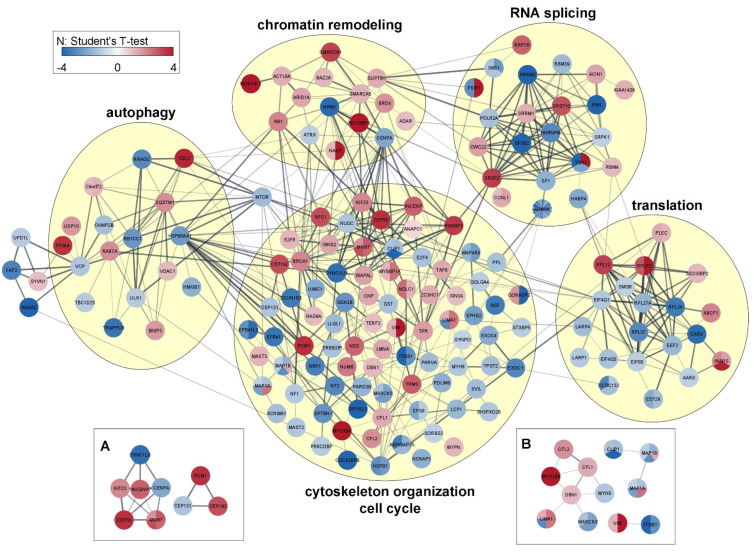
Network association among proteins with regulated phosphosites. The relations are grouped by biological processes. For each protein, the color indicates the direction of regulation for up or down and the relative value of that change is indicated with the color intensity bar taking into account the Student’s T-test difference intensity in the treatment group with HeberFERON, with respect to the untreated control group (log2 (Fold change)). Each circle represents one protein and the divisions denote the different phosphosites regulated and the magnitude of the regulation in the HeberFERON treatment group to untreated control according to the color intensity bar. Clusters of interest are presented in (**A**): cell cycle significant proteins and (**B**): cytoskeleton significant proteins. Gene symbols are shown.

The network interconnections among proteins with phosphosites regulated by HeberFERON were analyzed using STRING on the Cytoscape framework (Figure 5). For a better understanding, we grouped proteins by Biological Processes according to STRING classification. There are nodes with high degrees of more than 15 connections with other proteins, such as NPM1/B23 and CENPA in Chromating remodeling; CENPA as part of the Cell Cycle, proteins MYH9, MYO18A, BRCA1 or HSPB1/HSP27 in the cytoskeleton organization and VCP & HSP90AA1 acting at autophagy. In RNA splicing, translation and autophagy processes, there are highly connected clusters. Translation shows several 60S ribosomal subunits and tRNA ligases proteins with distinct regulations, as well as components of the initiation complex (EIF4G1_S1092, EIF4G3_S232) or the elongation complex related to MTOR-RPS6KA3 pathway (EEF2_T57, EEF2K_T348) decreasing phosphorylation. RPS6KA3 protein was downregulated and phosphosites controlled by this kinase (i.e., HSPB1_S82 and LCP1_S5) congruently decreased phosphorylation. MTOR occupies a centric position, interacting with several other proteins in processes such as autophagy (TSC2, RB1CC1, HSP90AA1, ULK1 and SQSTM1), cell proliferation (GSK3B, BRCA1), cytoskeleton organization/cell cycle or translation (not shown). AHNAK, with 39 phosphosites decreasing phosphorylation, is located in the frontier between RNA Splicing (interacting with SF1) and cytoskeleton organization (interacting with Periplakin (PPL), Golgi protein GORASP2 and the Ephrin type-B receptor 2 (EPHB2)). Interactions with MYH9, LIMA1, HSPB1/HSP27, EEF2 and ANNXA2 could also occur although these are not shown in this network. Other proteins with multiply regulated phosphosites (i.e., AHNAK2, ANAP12, Leprin beta 1 (PPFIB1), SNX29) are not connected in the main interaction network but most of the proteins, even those with no previous report of phosphosites or with no clear kinase association, are included.

## 4. Discussion

In this article, we present the first label-free quantitative proteomic and phospho-proteomic results after the treatment of the glioblastoma-derived cell line U-87 MG with HeberFERON for 72 h. 

Interferons are pleiotropic molecules with a recognized antiviral effect and antiproliferative outcome on cancer cells [1]. Type I and II combinations take advantage of their cross-talk and common pathways to achieve synergic effects [2]. Previous work at earlier times (6 h and 24 h) have shown the modulation of genes participating in transduction cascades and their control, antiviral effect, activation of several components of the immune system and translation machinery, apoptosis, cell cycle, the metabolic cellular process and others [28,29]. Kinetic series from 2 h to 96 h to study transcriptomic profiles after IFNα or IFNγ treatments in HUVEC found a marked increase in gene expression after 2 h of stimulation with a peak at 5 h, but a differential kinetic later [30]. After IFNα stimulation the expression of analyzed genes returned to baseline levels by 18 h, whereas it remained persistently up-regulated after stimulation with IFNγ, up to 96 h. Hall et al. also dissected IFNα or IFNγ biomarker responses in a human submandibular gland epithelial cell line in a time series experiment until 48 h, also reporting the delayed and persisting activation by Type II IFN [31]. A global proteome alteration induced by type I and II IFNs was studied in the human cell line MRC-5 at 4, 24 and 48 h. Differences in the changes induced by IFNα and IFNγ became evident upon prolonged exposure, where the effect was most apparent for repressed proteins, almost exclusively observed in response to IFNγ [32]. Studies with IFNγ stimulation of a melanoma cell line in a time courses experiment from 3 h to 72 h [33] showed a progressive increase in significantly regulated mRNA and miRNA; miRNAs only become connected to biological functions at very late time points (48 h and 72 h), indicating that they are activated by downstream regulators from the activated JAK/STAT signaling cascade. The presence of IFNγ in the formulation provides new properties to the IFNα and IFNγ-itself actions. 

The observation that HeberFERON effects, in preclinical xenografted models in mice (unpublished results) and in clinical protocols are not immediate [5], along with previous molecular elements, encourage us to investigate the action of this co-formulation at a later time. In pharmacodynamics studies in healthy volunteers, OAS and β2-microglobulin biomarkers were still highly detected in blood at 72 h [34]. A reduction of U-87 MG cell numbers to about 50% after HeberFERON treatment at IC50 dose was reported at 72 h [12]. 

IFN signaling starts with the binding of the IFNα and IFNγ to their receptors and follows the canonical JAK/STAT pathway, where STAT1 and STAT2 in association with IRF9 initiate the signal transduction cascade and different complexes translocate to the nucleus to activate the expression of genes encoding proteins involved in diverse processes. STAT1, STAT2 and IRF9 increased protein expressions with HeberFERON. Many interferon-stimulated genes from ISRE or GAS promoter sites can explain direct antiviral, innate immune and antiproliferative responses [35,36,37]. The increase of several classes I and II MHC molecules, proteins participating in MHC-peptide presentation, post-translational modifications, or proteasomes, explains the activation of adaptive immunity [38]. Activation of IFIT3 [39], 2′-5′ OAS and RNase L [40], PKR [41] and IRF9 [42] could also contribute to the antiproliferative effect on U-87 MG cells. In glioblastoma-derived T98G cells, IRF9 overexpression enhanced the antiproliferative activity of IFN-α2c in resistant cells and induced apoptosis [42]. All these biomarkers are similarly activated at transcriptomic levels [12] in correspondence to previous studies with IFN combinations [28,29]. 

Although HeberFERON is a co-formulation of Type I and II IFNs, their very carefully selected proportion could give advantages in exerting longer time synergic effects [5]. Not only the crosstalk between both IFN cascade signaling [2] but the IFN signaling feedback controls, including receptor recycling [43], could better explain the higher and longer effects of HeberFERON compared to IFNs type I or II independently [5]. This experiment gives new clues. Upon IFNα stimulation, PKD2 is recruited to IFNAR1, phosphorylated by TYK2, which in turn phosphorylates IFNAR1 on S535 and S539 residues. These phosphorylations are important in the internalization of the IFNAR complex. After internalization and arrival in early endosomes, degradation in lysosomes is used for IFNAR1 to control signaling. Here, we found the site not previously described IFNAR1_S426 with increased phosphorylation. Nevertheless, it is suggested that IFNAR2 is recycled back to the plasma membrane by a retromer complex [43]. This complex is assembled by a first trimer sub-complex, made of vacuolar proteins sorting-associated protein 35 (VPS35)-VPS29-VPS26, that binds the early endosome phosphoinositide PI(3)P through a domain present in sorting nexin (SNX) and, together with the small GTPase RAB7A, assembles the second sub-complex of the retromer. IFNAR2 can interact with RAB35 and VPS26A, VPS29 and VPS35. In our data VPS26B and RAB35 decreased and increased protein expressions, respectively. Phosphosites VPS26A_S2 and S268/S330/S344 on SNX29 decreased phosphorylation; RAB7A_S72 increased phosphorylation. Only the phosphorylation in RAB7A has been associated with LRRK1 to regulate cargo-specific trafficking [44]. Controlling the residency time of internalized IFNAR complex in the endosome, the retromer is directly implicated in the fine tuning of JAK-STAT signaling duration and downstream transcription outputs thus the long-term effects of HeberFERON could be explained by the persistence of receptor-bound IFN-α2 inside endosomes and IFN signaling from this compartment for days, even when the negative regulators ISG15 or USP18 were missing [37]. The regulation of components contributing to IFNAR2 recycling and the new phosphosite on IFNAR1 could explain part of the distinctive HeberFERON effects [5]. 

In a previous microarray experiment, the cell cycle is highly impacted by HeberFERON with a decrease in several mitotic prometaphase to anaphase players, mainly participating in the spindle checkpoint and proteolytic degradation by APC/C-CDC20, which could explain the G2/M arrest after 24 h [12]. Although differences in genes and proteins expressions were temporal, we expected that the importance of this biological process as a target was also unveiled in this experiment. The histone H3 variant centromere protein A (CENPA) specified accurate segregation of sister chromatids during mitosis [45]. Phosphorylation of CENPA on S17 and S19 by CDK2 is important for chromosome segregation and unphosphorylated variants result in mitotic errors [45]. HeberFERON diminished the phosphorylation of both sites (S17 and S19) on CENPA, a protein highly connected to the phospho-proteomic network. INCENP ensures the correct chromosome alignment and segregation, as a scaffold protein regulating the chromosomal passenger complex localization, and it is required for chromatin-induced microtubule stabilization and spindle assembly [46]. INCENP_S420 and INCENP_S424 increase phosphorylation in mitosis [47], but HeberFERON increased the phosphorylation of INCENP_S421. This site has been described in PSP phosphorylated by CDK1 with no biological function. Other members of this hetero-tetrameric complex, Aurora kinase B and Survivin, decreased gene expression at 72 h. NIMA Related Kinase 9 (NEK9), which regulates spindle organization and cell cycle progression [48], also decreased protein levels. Increased phosphorylation of unreported phosphosites in the APC/C component ANAPC1_S223 and the kinesin KIF23_S422 [46] occurred with HeberFERON. Significant gene expression decreases for CENPA, INCENP, APC/C-CDC20 and KIF23 with HeberFERON to individual IFNs were observed in the microarray experiment, reinforcing their roles in the observed G2/M arrest [12]. Proteins participating in different cell cycle phases were regulated. MKI67 is required to maintain mitotic chromosomes dispersal, thus it is present at the highest level in the G2 phase and during mitosis. We found three phosphosites (MKI67_S584,_S2527 and _S2707) with no biological function described increased phosphorylation [49]; only S584 is predicted to be a CDK1 substrate by NetworKIN. Otherwise, RB1 promotes G0-G1 transition after phosphorylation at both S807 and S811 by CDK3/cyclin-C [50]. HeberFERON treatment increased phosphorylation of the first, while S811 was not detected. The histone chaperone NPM1 has multiple roles in ribosome assembly and transport, cytoplasmic-nuclear trafficking, regulation of p53 and centrosome duplication [51]. NPM1_T219 together with T199, T234 and T237 are phosphorylated before mitosis by the CDK1-cyclin B complex; lack of phosphorylation or NPM expression result in spindle checkpoint activation due to severe mitotic defects [52]. After HeberFERON treatment, NPM1_T219 diminished phosphorylation. This protein has a high degree of connection in the phospho-proteome network. The findings regarding regulation of MKI67, RB1 or NPM1 deserve future studies.

IFNs could also achieve an antiproliferative response through non-canonical signaling including PI3K/AKT/mTOR and MAPK cascades and, in correspondence, proteomic changes showed modulation of several kinases. ERK1 and ERK2, from the MAPK protein family, can impact cell cycle progression, cytoskeleton organization and mRNA translation [53]. mTOR impacts translation, cytoskeleton and autophagy [54]. The protein kinases (PK) ABC family also participates in signaling leading to proliferation control [55]. In the proteomic data PKA regulatory subunits PRKAR2A and PRKAR2B and PKCα (PRKCA) decreased expressions; several sites controlled by these kinases diminished phosphorylation levels after IFNs treatment.

The Ser/Thr kinase mTOR cascade is commonly up-regulated in cancer due to loss of the tumor suppressor PTEN, as in U-87 MG [56]. PI3K/AKT, as well as the mitogen-activated protein kinase (MAPK) pathways engage mTOR. In an apparent contradiction, the PI3K/mTOR axis is necessary for the induction of apoptosis after the treatment of tumor cells with IFN-α [4]. Here we showed that mTOR occupies a centric position in the phospho-proteomic network with functional interaction with components of the translational machinery, cytoskeleton and autophagy. Interesting changes in proteins participating in these processes are commented on. 

HeberFERON decreased the phosphorylation of mTOR_S1261 which is mediated by several kinases (PKCα, GSK3β, RAF1, ROCK1, or AKT1). This TSC/Rheb signaling promotes phosphorylation, then promotes mTORC1-mediated autophosphorylation and substrate phosphorylation to stimulate cell growth [57,58]. This change in mTOR phosphorylation status, probably together with the increase of phosphorylation in unreported phosphosite TSC2_S1743, could affect downstream substrates, depending on S1261 phosphorylation. Phosphosites EEF2K_T349 and EEF2_T57 diminished their phosphorylation, which then promotes protein synthesis. Calmodulin-dependent eukaryotic elongation factor 2 kinase (eEF-2K) _T349 autophosphorylation appears to control the catalytic output of active eEF-2K, contributing more than five-fold to its ability to phosphorylate eEF-2_T57 [58]. How this is part of a cellular rescue response should be investigated. mTORC1 regulates mRNA translation by phosphorylation of components of the initiation translation complex. EIF4G1_S1092 and EIF4G3_S232 phosphosites with unknown functional consequences decreased their phosphorylations; this could change the translatome stoichiometry as part of the mechanism derived from mTOR influence [59]. Changes in the phosphorylation status of EIF5B and 60S ribosomal proteins could complement this effect. In this same signaling branch, the ribosomal protein S6 kinase alpha-3 (gene RPS6KA3) that regulates translation through RPS6 and EIF4B phosphorylation was found with decreased protein expression. 

The cytoskeleton is a complex network of microtubules, actin and intermediate filament that contributes to cell growth and migration and mTORC2 controls the actin cytoskeleton. mTORC2-associated interactome showed actin-binding proteins and microtubule-associated proteins significantly changed depending on mTORC2 activity level in glioblastoma, including Myosin-9/MYH9, Plectin/PLEC and MAP1B [60]. Microtubule-associated proteins (MAP)1 family are associated with both microtubules and actin and their phosphorylation regulates the filamentous cross-bridging between microtubules and other skeletal elements [61]. MAP1A and MAP1B showed several not described sites or with no associated biological function that diminished (MAP1A _T854/S1526/S2260 and MAP1B_S1512/S1881/S1915/S1917) and increased (MAP1A_S1398, MAP1B_T1788) phosphorylation. Deficiencies in expression or lack of phosphorylation of MAP1B by GSK3β led to abnormalities during brain development. MAP1B_T1788, MAP1B_S1881 and MAP1B_S1915 are predicted substrates for GSK3β but also CDK1, CDK5, GSK3α kinases in NetworKIN. Additional GSK3β regulated sites on cytoskeleton proteins (CLIP1_S200 and CLIP2_S207) diminished phosphorylation. The proline-directed protein kinase GSK3β [62] looks like another important hub in the HeberFERON action and it is functionally related to mTOR signaling [63]. This kinase is inhibited by phosphorylation of GSK3β_S9 but, instead, we found a decreased phosphorylation on GSK3β_S25 with unknown biological relevance or responsible kinases [62]. Other actin-binding proteins, highly connected in the phospho-proteome network, were regulated by phosphorylation after HeberFERON treatment including MARCKS, CFL 1 and 2, Eplin (LIMA1), MYH9 and MYO18A although most of the phosphosite changes have unknown effects [64]. Eplin coordinates actin and myosin dynamics throughout cell division by increasing the number and size of actin stress fibers; it is phosphorylated at the C-terminal region by ERK1/2 which reduces its association with F-actin and contributes to actin filament reorganization and cell motility enhancement [65]. After HeberFERON treatment, three sites increased phosphorylation in this protein (S210, S363 and S610) and one decreased (S370), but only S362 is reported as being phosphorylated by ERK1, with a downstream effect related to cytoskeleton organization and cell motility. Compensatory and synergistic effect of double phosphorylation at Eplin_S362 and S604 residues has been reported and it is potentially possible that other residues may also be involved in the regulation of Eplin [65]. The increased phosphorylation of S3 in CFL1 and CFL2 [66], inactivates these proteins in their role in the normal progress of mitosis and cytokinesis. MARCKS is mostly regulated by PKC [67] and showed a decreased phosphorylation on MARCKS_S29 regulated by PRKCG and PRKCD; but also decreased phosphorylation MARCKS_S27 is regulated by CDK2 and ERK2 and the unreported MARCKS_S63. Moreover, class-III intermediate filament protein Vimentins (VIM) is highly connected in the phospho-proteome network and showed two sites (VIM_S339 and VIM_S420) that increased phosphorylation after HeberFERON. Although none of these sites has a downstream biological function reported, NetworKIN predicts the phosphorylation of S420 by RAF1 and ROCK1. In U251 glioblastoma-derived cell line interaction between VIM and GSK3β was demonstrated with implications in cell migration; HeberFERON modified phosphorylation on both proteins [68]. The small heat shock protein HSP27 (HSPB1) plays a role in stress resistance and actin organization functioning as a molecular chaperone and in maintaining denatured proteins in a folding-competent state [69]. HSP27 phosphorylation impairs its ability to protect against oxidative stress but HeberFERON decreased phosphorylation in S82 and S86. S82 phosphorylation by AKT1, MAPKAPK2 and P70S6KB plays a role in cell growth and cytoskeletal reorganization, while S86 phosphorylation has an unknown biological function. Undoubtedly, cytoskeleton reorganization plays a role in molecule movement in the cell and their components, highly interconnected with other proteins, impact on cell cycle, translation, autophagy, DNA repair, chromatin organization, or splicing processes, contributing to limiting cellular proliferation. 

The third process that mTORC1, together with GSK3β, regulates downstream of the PI3K/AKT pathway is autophagy [58]. Autophagy is a crucial lysosomal-dependent cellular degradation process for cell survival under extreme conditions, preserving cells from further damage [70]. However, autophagy also plays a death-promoting role, as a tumor suppressor mechanism in cancer and, together with apoptosis, could contribute to limiting cancer cell proliferation. In response to starvation or after a pharmacological blockade, the mTORC1 affects the phosphorylation of sites in ULK1/2, Atg13 and RB1CC1 leading to autophagy induction; in this process, the HSP90-CDC37 chaperone complex selectively stabilizes and activates ULK1. Phosphorylation sites on the highly connected HSP90A (HSP90AA1) can affect the interaction between HSP90 and its co-chaperones and clients, many of which are protein kinases [71]. The ATPase enzyme VCP is also involved in the maturation of ubiquitin-containing autophagosomes and the clearance of ubiquitinated protein by autophagy [72]; this protein shows several connections in the phospho-proteome network and the decrease of phosphorylation in VCP_S13. The role of HeberFERON in autophagy is demonstrated by the decreased phosphorylation on phosphosites RB1CC1_S516, ULK1_S623, HSP90A_S315 and VCP_S13 although their exact biological functions are unknown. Selective autophagy plays a regulatory role in the cell and, in cooperation with the ubiquitin-proteasome system, acts as an ON-OFF switch of cell cycle progression via controlling the stability of several regulators such as CDKs, CKIs and checkpoints; these regulators can also regulate autophagy [73]. SQSTM1 is one of the receptors participating in selective macro-autophagy recognizing CDK1, cyclin D1 and p27 and recruiting them into autophagosomes. Their relationship with CDK1-Cyclin B1 has positioned SQSTM1 as a node for the control of cell survival and cell transit through mitosis [74]. Here, SQSTM1_S272, phosphorylated by CDK1 and MAPK P38 Delta, increased its phosphorylation. Interestingly, microarray data showed a decrease in cyclin D1 and cyclin B1 gene expression [12]. Macro-autophagy is involved in the replication stress response and DNA repair pathways [75]. Several proteins participating in the DNA repair process and controlled by CDK1 and CDK2 [BRCA1, UIMC1, RAD9A and RFC1] modified phosphorylation levels after HeberFERON treatment. Of note, it is the highly interconnected E3 ubiquitin-protein ligase BRCA1 that showed increased phosphorylation in BRCA1_S114 which is important for fork and nascent DNA strand protection [76]. The real implication of autophagy as a survival mechanism or as a cellular death mechanism needs to be further elucidated. 

Lastly, we found proteins with scaffolding properties that showed several sites with decreased phosphorylation, including AHNAK (39 sites), AHNAK2 (18 sites), AKAP12 (eight sites) and Liprin beta1 (PPFIBP1, seven sites). AHNAK could be a regulator of PKCα activity [77] that was found diminished in the proteome. In confluent cells, it is phosphorylated by protein kinase B (PKB)/AKT which leads to the translocation of AHNAK to the plasma membrane, where it forms a protein complex with Annexin 2 (ANXA2)/S100A10 and the actin cytoskeleton contributing to cell–cell contacts. Together with the multiplied phosphosites diminishing phosphorylation on AHNAK, HeberFERON also decreased the phosphorylation of ANXA2_S26 with a probable effect on actin remodeling. Treatment with PI3K inhibitor, upstream of PKB/AKT, resulted in decreased levels of phosphorylated AHNAK located in the nucleus. Decreased phosphorylation of a few sites (S4903, S5279, S5321, S5854 and S5857) is possibly modified by PKC kinases, while AHNAK_S5763 is predicted to be regulated by CDK1. The future implication of this protein in cell growth through interferon non-canonical signaling should be studied. 

U-87 MG as a tumoral cell promotes the proliferation through different signaling pathways (MAPKs, PI3K/Akt/mTOR, PKA and PKC) where crosstalk between them is common in cell regulation. HeberFERON acts over most of these signaling and fine-tunes the biological responses. The modulation of cell cycle components, firstly demonstrated at the gene level, looks like an important mechanism also at the protein level and could explain the antiproliferative effect of the co-formulation after 72 h of treatment. Although there are 27.3% of cells arrested in S/G2/M in comparison to 8.9% in the control cells at that time point, working at the IC50 dose, it is possible to have a picture of changes occurring in those cells still alive. The relevance of cell cycle arrest was also observed in GBM clones in cell cultures with a high antiproliferative response to HeberFERON (unpublished data). The influence of this IFN combination on translation and RNA processing will also contribute to maintaining the antiproliferative effect during the experimentation. HeberFERON can also contribute to diminishing cell numbers by death mechanisms such as apoptosis and autophagy. Both processes appeared enriched in phospho-proteome data. Time course future experiments could better describe a sequence of events and transcript and protein relationships in the mechanism of action of HeberFERON. Investigations using glioblastoma clones and animal models could even strengthen these findings. 

An interesting finding in our phospho-proteome experiment is that most of the substrate phosphorylation increases were provoked by CDK1/CDK2 and ERK1/2, kinases with a recognized role in cell cycle progression and survival pathways. These two cascades control cell proliferation under very strict regulation including positive and negative feedback loops and reciprocal regulation, which promote oscillations and dynamism necessary to adjust regulation [78,79]. This fine-tuning could lead cancer cells to arrest or rescue outcomes, using mechanisms such as autophagy or DNA repair. Our description of the novel or barely studied phosphosites opens new possibilities for experimentations and hypotheses. We could then speculate that by changing the phosphorylation status of a variety of proteins participating in biological processes related to survival and death, HeberFERON moves the balance through an arresting response. 

## 5. Conclusions

In this paper, we obtained for the first time answers about the main mechanism used by HeberFERON in U-87 MG to achieve an antiproliferative effect, through proteomic and phospho-proteomic data (Figure S2). Together with direct antiviral and immune system stimulation mechanisms, IFNα/γ co-formulation highly impacts the cell cycle and also the cytoskeleton organization, translation and RNA processing, autophagy and DNA repair using several signaling pathways and highly interconnected phosphoprotein hubs. mTOR is a hub occupying a very centric position that impacts cytoskeleton organization, translation and autophagy processes. IFN canonical (JAK/STAT) and non-canonical pathways converge to kinases such as CDK1/CDK2, ERK1/2, PK ABC, mTOR and GSK3 that regulate an enormous number of sites, many of them novel or with undescribed biological functions. Future investigations of these phosphosites could clarify their relevance for the HeberFERON mechanism but also their contribution to kinase-mediated regulation. Furthermore, the study of some changes will elucidate their influence on the antiproliferative effect or rescue response at 72 h, but earlier and later time course studies could complement that answer. Although this study was carried out in a cell line, our findings contribute to the understanding of how HeberFERON could exert pleiotropic effects and succeeds with a cellular antiproliferative response in glioblastoma. Evaluation of some described biomarkers during the treatment of GBM-bearing patients would underscore our findings. 

## Figures and Tables

**Table 1:** Principal substrates and phosphosites regulated by kinases following KSEA analysis. Phosphosites emphasized in bold letters were also obtained from KEA2 and/or iPTMNet analysis. Phosphosites increasing phosphorylation are in red and decreasing phosphorylation is in blue. Gene symbols and the position of phosphosite in each protein are shown.

Kinases	Representative Sites	Kinases	Representative Sites
**CDK1**	**AHNAK_S5763** , BRCA1_114, CCNL1_S342, **CEP55_S428**, **DUT_S99**, INCENP_S421, MAP1B_T1788, MKI67_S584, **NPM1_T219** , **RAD9A_S328**, RANBP2_T799, **RB1_S807**, **RFC1_T506**, SIN3A_S860, SMARCA5_S116, **SQSTM1_S272**, TCOF1_S583, TFDP1_S23	**PRKCA**	AHNAK_S4903, AHNAK_S5321, AHNAK_S5857, AKAP12_S612, **ANXA2_S26**, **C5AR1_S334** , **CLIP1_S204**, EIF5B_S222, ITGB1_S785, MARCKS_S29, MTOR_S1261, NF2_S13, SPTBN1_S2358, UFD1L_S299,
**CDK2**	BRCA1_114, BRD4_S470, CENPA_S19, COIL_S566, COIL_T303, HIST1H1B_T11, HIST1H1B_T138, **MARCKS_S27**, NPM1_T219,**RAD9A_S328**, **RB1_S807**, **RPL12_S38**, TCOF1_S583, TERF2_S365, TFDP1_S23, TPR_T1677	**PRKCB**	AHNAK_S4903, AHNAK_S5279, AHNAK_S5321, AHNAK_S5854, AHNAK_S5857, AKAP12_S612, **ANXA2_S26**, **C5AR1_S334** , CLIP1_S204, DR1_S105, EIF5B_S222, **HSPB1_S86**, NF2_S13, PARVA_S28, PCNP_S87, RBM39_S117, SPTBN1_S2358, UFD1L_S299
**AURKA**	CEP55_S428, INCENP_S421, MKI67_S584	**PLK1**	CLIP1_S195, CLIP1_S200, CLIP2_S204, NUDC_T145
**MAPK3 (ERK1)**	BAG3_S386, RB1_S807, SQSTM1_S272	**RPS6KA3**	**HSPB1_S82**, **LCP1_S5**
**MAPK1 (ERK2)**	**CEP55_S428**, RANBP2_T799, RB1_S807, TPR_T1677, MARCKS_S27	**PRKDC**	ATRX_S1348, FASN_S2198, FASN_S2236, LYST_S2166
**CDK7**	LEO1_S162, MTA1_S576, TAF3_S183,	**PRKACA**	**LCP1_S5**, **HSPB1_S82**, **PPP2R5D_S573**, **SPTBN1_S2358**, TAGLN2_S163,
**CDK3**	**RB1_S807**, TFDP1_S23	**PRKCE**	AKAP12_S612, MARCKS_S29, NF2_S13
**CDK4-CDK6**	CCNL1_S335, **RB1_S807**	**EIF2AK2**	MTOR_S1261, NPM1_T219
**RAF1**	MTOR_S1261, NF1_S2188, RB1_S807, VIM_S420	**AURKB**	CLIP1_S195, CLIP1_S200, NPM1_T219, MARCKS_S29
**ROCK1**	CEP55_S428, CFL1_S3, DPYSL2_T514, MTOR_S1261, VIM_S420	**PKD1**	HSPB1_S82, HSPB1_S86
**GSK3B**	CLIP1_S200, CLIP2_S207, **DPYSL2_T514**, **HIST1H1B_T11**, HIST1H1B_T138, MAP1B_T1788, MAP1B_S1881, MAP1B_S1915, MTOR_S1261	**AKT1**	FASN_S2236, **HSPB1_S82**, MTOR_S1261, NF2_S13, NF1_S2188,
**CDK5**	**DBN1_S142**, DPYSL2_T514, MAP1B_T1788, MAP1B_S1881, MAP1B_S1915, **RB1_S807**	**PRKAA2**	EEF2K_T348, FASN_S1411, FASN_S2236, MTOR_S1261, SCD_S198

**Table 2:** Substrates and regulated phosphosites not described before (No PTM) or described with no kinase-associated (PTM w/No Ez) following iPTMNet analysis. Phosphosites with increased phosphorylation are in red and those with decreased phosphorylation are in blue. Gene symbols and the position of phosphosite in the proteins are shown. They are grouped by biological process.

PTM w/No Ez		No PTM		PTM w/No Ez		No PTM	
**Chromatin remodeling**				**RNA Splicing**			
SMARCA5	**S116**	SMARCA1	**S116**	AHNAK	**S1010/S1123/** **S1445/S2560/** **S3360/ S3409/S3412/ S4520/** **S4870/S4933/** **S5031/ S5289/** **S5332/S5400/** **S5430/ S5530/** **S5589/S5620/** **S5641/S5731/** **S5735/S5749/** **S5752/S5830/** **S5851/ S5857/S6580/** **S6910/** **T2470/T3716/** **T4100/T5824/** **T590**	HNRNPM	**S481**
ATRX	**S1348/** **S1352**			CCNL1	**S335/S338**	SF1	**S14**
**Autophagy**				IWS1	**S224/S248/** **S250**	SRSF10	**S234**
CHMP2B	**S199**			PNN	**S690/S694/** **S695**		
HSP90AA1	**S315**			SRRM2	**S1972/T1974**		
PDIA4	**S470**			SRSF2	**S189/S191**		
RAB7A	**S72**						
RB1CC1	**S1222**						
**Cytoskeleton organization/Cell Cycle**				**Other processes**			
GORASP2	**S409**	CEP192	**S1355**	AKAP11	**T1485**	ADAR	**S624**
GSK3B	**S25**	GOLGA4	**S266**	AKAP12	**S1251/S248/S598/S660/S806/T608**	AHNAK2	**S248/S397/** **S593/S746/** **S823/S1153/** **S1318/** **S1813/** **S1978/** **S2896/** **S2896/** **S3217/ S3946/** **S4534/** **S4610/** **S4612/** **S4862/** **S4862/ S4866/** **S4957/** **S223**
MAP1B	**S1512/** **S1881/ S1915/** **S1917**	GORASP2	**T347**	AKAP2	**S361**	AKAP12	**T523**
MARCKS	**S29**	KIF23	**S422**	ATF7IP	**S445/S673**	ANAPC1	**S223**
MYH9	**S1340**	KIF26B	**S1608**	CAMK2D	**S490**	BCLAF1	**Y381/S420**
MYO18A	**T722**	LIMA1	**S210/** **S363** **/ S370/** **S610**	COIL	**S566/T303**	CSNK1E	**S66**
MYO9B	**S1972**	LMNA	**S598**	FASN	**S1411/S2198/** **S2236**	IFNAR1	**T328**
		MAP1A	**T854**/ **S1398** **/S1526/ S2260**	GLI2	**S234**	PPFIBP1	**S449/S573/** **S579/** **S595/S788/** **S995** **/S997/ S1001**
		MARCKS	**S63**	PTPN14	**S594**	PRKD1	**S245**
		**MKI67**	**S2527/** **S2707**	RAB3GAP1	**S537**	RAB28	**S2/S4**
		MAP4K4	**T810/** **S875/** **S934**	RABEP1	**S410**	RIOK2	**S332**
		NOLC1	**S547/** **T619**	RIOK2	**S337**		
		PCM1	**S1188**	SNX29	**S268/S330/S344**		
		SYNPO	**S894**				
**Translation**							
AARS	**S403**	LARP4	**S651**	LARP1	**T526**		
EIF4G1	**S1092**			RPL24	**T83**		
EIF4G3	**S232**			RPL27A	**S68**		
EIF5B	**S214**			RPL37	**S97**		
PLEC	**S1435**			RPLP2	**S79**		

## Data Availability

All data of proteomic and phospho-proteomic results generated during this study are included in this published article and supplementary information files. Any additional requested information would be available from the corresponding author upon reasonable request.

## Supplementary Materials

The following supporting information can be downloaded at: https://www.mdpi.com/article/10.3390/cells11244068/s1. Figure S1: Network representation of the interconnected elements in the proteome data from STAT1/STAT2. a: components of the antiviral response, b: components of the MHC and proteasome. Color intensities represent protein changes in treated samples compared to cell control, as indicated in the bar intensity color according to Student’s *t*-test Difference LFQ intensity HF_LFQ intensity CC (log2 (Fold change)) from Table S2: Bisogenet (v.3.0.0) plugin was used on the Cytoscape framework (v.3.8.2). HF: HeberFERON; CC: cell control. Figure S2: General pathway and biological processes regulated by HeberFERON in U-87 MG as it is shown in proteomic and phospho-proteomic data. The figure was created with BioRender (biorender.com). Table S1: Results of Phospho-proteomic experiment showing phosphosites significantly changing. For each phospho-peptide the table indicates in this order: Amino acid, Position, Charge, Multiplicity, Score, Delta score, Mass error [ppm], -Log T-test *p*-value Intensity HF_Intensity CC (log10), Student’s *t*-test Difference Intensity HF_Intensity CC (log2(Fold change)), Protein (Uniprot ID), Protein names, Gene names, Sequence window (16 amino acids upstream and downstream from the site), # (counting). HF: HeberFERON; CC: cell control. Table S2: Results of a proteomic experiment with proteins differentially expressed by HeberFERON. For each protein, the table indicates in this order: Peptides (in the identification), Unique peptides, Sequence coverage [%], Mol. weight [kDa], Q-value, Score, -Log Student’s *t*-test *p*-value LFQ intensity HF_LFQ intensity CC (log10), Student’s *t*-test q-value LFQ intensity HF_LFQ intensity CC, Student’s *t*-test Difference LFQ intensity HF_LFQ intensity CC (log2(Fold change)), Protein IDs, Protein names, Gene names, # (counting). HF: HeberFERON; CC: cell control. Table S3: Enrichment analysis of Pathways in proteomic data using the Toppfun tool. Pathways in Toppfun, *p*-value, Genes Symbols. Table S4: Enrichment analysis with data from proteomic using DAVID as a tool and different backgrounds (identified proteins or *Homo sapiens*). Columns represent Category and Term (in databases), Count (number of proteins), %, *p*-Value, Genes, Fold Enrichment and Bonferroni, Benjamini, FDR corrections. Similar color for similar categories is used, highlighting in yellow the coincidences between analyses with different backgrounds. Table S5: Enrichment analysis with data from phospho-proteomic using DAVID as a tool and different backgrounds (identified proteins or *Homo sapiens*). Columns represent Category and Term (in databases), Count (number of proteins bearing changing phosphosites), %, PValue, Genes, Fold Enrichment and Bonferroni, Benjamini, FDR corrections. Similar color for similar categories is used, highlighting in yellow the coincidences between analyses with different backgrounds. Table S6: Analysis of kinase motifs using the Motif-All algorithm in PSP. Motif, ZScore, *p*-Value, Fold Change, Foreground Matches, Foreground Size, Background Matches, Background Size, Kinases.
