# Supplementary material for: Proteomics and Phospho-Proteomics Profiling of the Co-Formulation of Type I and II Interferons, HeberFERON, in the Glioblastoma-Derived Cell Line U-87 MG
Source: Cells. 2022 Dec 15;11(24):4068. doi: 10.3390/cells11244068 (PMC9776974; doi:10.3390/cells11244068)
Supplement: Supplementary file 1 [file cells-11-04068-s001.zip › Figure 1S Resubmission.pdf]

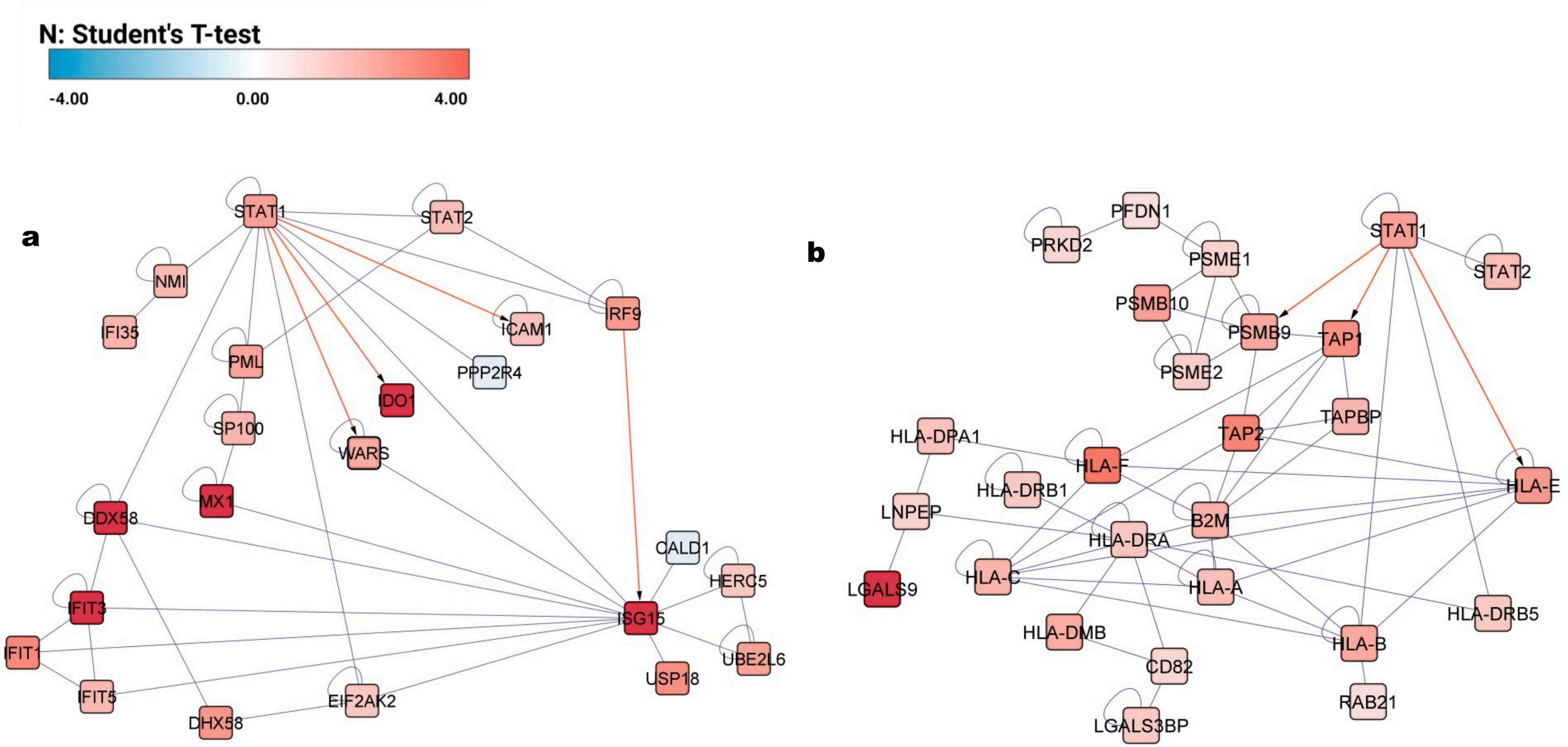

Figure 1S. Network representation of the interconnected elements in the proteome data from STAT1/STAT2. a: components of the antiviral response, b: components of the MHC and proteasome. Color intensities represent protein changes in treated samples compared to cell control, as indicated in the bar intensity color according to Student's T-test Difference LFQ intensity HF_LFQ intensity CC (log2 (Fold change)) from Table 2S. Bisogenet (v.3.0.0) plugin was used on the Cytoscape framework (v.3.8.2). HF: HeberFERON; CC: cell control.
