# Supplementary material for: Proteomics and Phospho-Proteomics Profiling of the Co-Formulation of Type I and II Interferons, HeberFERON, in the Glioblastoma-Derived Cell Line U-87 MG
Source: Cells. 2022 Dec 15;11(24):4068. doi: 10.3390/cells11244068 (PMC9776974; doi:10.3390/cells11244068)
Supplement: Supplementary file 1 [file cells-11-04068-s001.zip › Figure 2S.pdf]

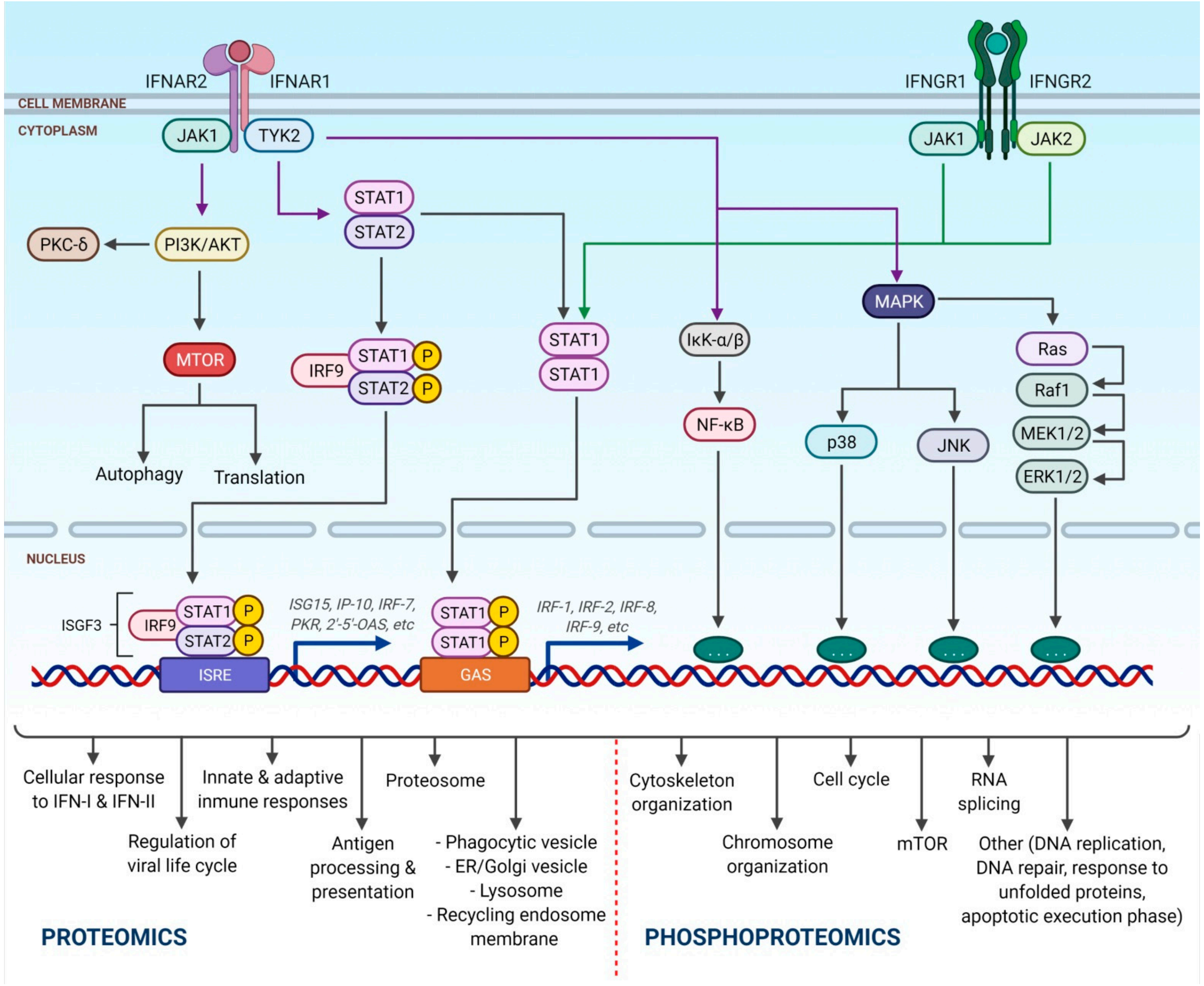

Figure 2S. General pathway and biological processes regulated by HeberFERON in U-87 MG as it is shown in proteomic and phosphoproteomic data. The figure was created with BioRender (biorender.com).
